# Unilateral Hypertrophy of Tensor Fascia Lata: A Case Report

**DOI:** 10.7759/cureus.32463

**Published:** 2022-12-13

**Authors:** Layann Y AlGallaf, Yasser N Asiri, Fahad A AlDawsari, Fouad I Al Adel

**Affiliations:** 1 College of Medicine, Imam Abdulrahman Bin Faisal University, AlKhobar, SAU; 2 Department of Musculoskeletal Radiology, King Fahad Specialist Hospital, Dammam, SAU

**Keywords:** muscle pseudohypertrophy, thigh mass, soft-tissue tumor, tensor fascia lata (tfl), hypertrophy

## Abstract

Hypertrophy of the tensor fascia lata muscle is a rare entity that may be observed in patients presenting with a palpable mass in the antero-lateral aspect of the proximal thigh area. Imaging confirms the diagnosis of such a rare entity. This case report highlights a case of isolated hypertrophy of the tensor fascia lata muscle with no identifiable etiology. Biopsy and surgical intervention were not needed due to the interval stability throughout a three-year period. The orthopedic oncology team reassured the patient and followed up the patient annually.

## Introduction

Palpable lump in the anterolateral aspect of the thigh has broad differential considerations including neoplastic, benign entities, and tumor mimickers. Isolated hypertrophy of the tensor fascia lata (TFL) muscle is considered in the latter category. Tensor fascia lata hypertrophy is a rare benign clinical entity that may be considered a differential in patients who complain of swelling and pain in the anterior thigh [1,2]. The TFL is a skeletal muscle situated over the anterior-lateral aspect at the thigh's root. The insertion site is along the anterior section of the anterior-superior iliac spine, in the lateral margin of the iliac crest, and the deep surface of the fascia lata is where more superficial fibers are located [1]. The TFL muscle aids in stabilizing the pelvis and stops it from tipping to one side when depending on one limb. It also helps in hip abduction and internal rotation alongside the gluteus medius and minimus muscles [3]. Moreover, it functions as an additional knee flexor and aids in stabilizing the knee in extension because of its continuity with the iliotibial tract [3]. Imaging with MRI is the best modality for assessing soft tissue tumors [4].

## Case presentation

**Figure 1:**
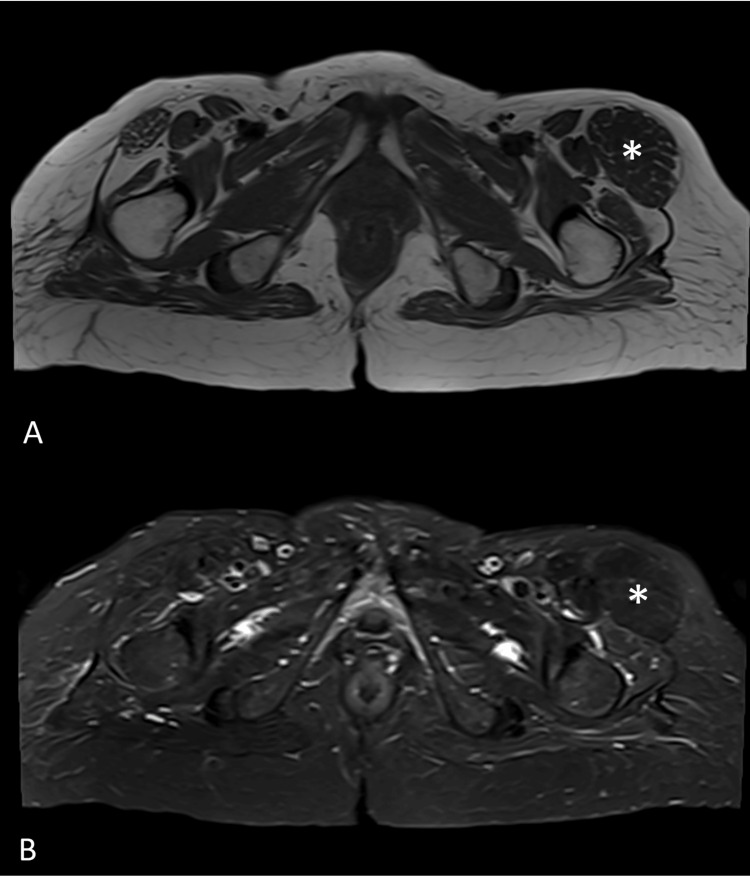
Axial T1 and T2 Weighted Images (A) : Axial T1 weighted image at the level of the proximal thighs demonstrates a diffuse enlargement of the left tensor fascia lata muscle (white asterisk) with preserved muscle fibers. No significant fatty replacement. (B): Axial T2 with fat saturation demonstrates the enlarged left tensor fascia lata muscle with no significant muscle edema.

**Figure 2:**
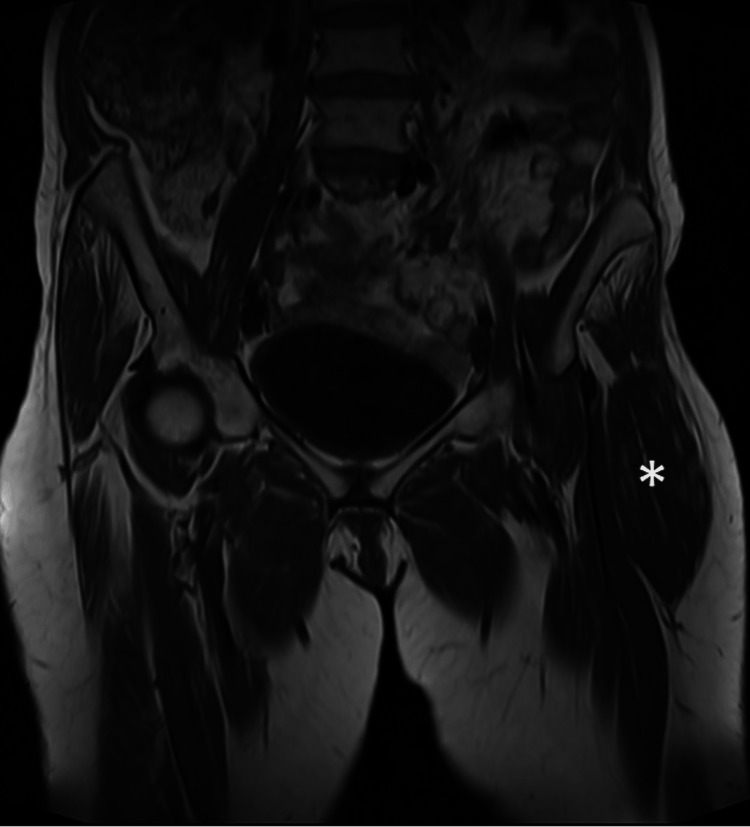
Coronal T1 Weighted Image Coronal T1 weighted image of the pelvis shows the asymmetrical enlargement of the left tensor fascia lata muscle (white asterisk).

A 67-year-old female known case of type 2 diabetes mellitus presented to our hospital complaining of left hip swelling. She noticed the swelling three months prior to her presentation. It was associated with mild pain. The patient has no numbness or weakness or any constitutional symptoms. Physical examination revealed a mass over the TFL, and it was noted to be deep to the fascia. The mass was mobile, oval-shaped, soft to firm with no tenderness. The patient has no history of trauma or surgery in that area. Axial MR T1 nonfat saturated and T2 fat-saturated images (Figures 1A, 1B) and coronal MR T1 nonfat saturated image (Figure 2) were performed in our 1.5 Tesla Siemens Sola machine. 

The images (Figures 1, 2) demonstrated no muscle fatty replacement or edema and no abnormal enhancing mass. MRI images showed an isolated enlargement of the left tensor fascia lata muscle with preserved muscle architecture and fibers. No aggressive soft tissue masses are seen. Follow-up MRI studies performed after two and three years demonstrate interval stability of the muscle hypertrophy. The overall findings are compatible with unilateral hypertrophy of the tensor fascia lata.

The patient was monitored by the orthopedic oncology team and was reassured after confirming stability for three years. A yearly follow-up regimen is currently in place for the patient.

## Discussion

Muscle hypertrophy involves an increase in muscle volume based on an increase in the number and size of muscle fibers. In contrast, in pseudohypertrophy, the increase in volume occurs because of the infiltration of fat and connective tissue into the muscle belly [5]. Imaging studies, specifically MRI, are helpful in the differentiation between muscle hypertrophy and pseudohypertrophy by assessing the amount of fatty infiltration [6].

Tensor fascia lata muscle hypertrophy is a rare pathology, with only a few cases published in the literature. Imaging with MRI studies would demonstrate diffuse enlargement of TFL muscle, with preservation of its morphology and signal characteristics [1].

A case report conducted by Cedric De Clercq et al., has found a patient with bilateral tensor fascia lata hypertrophy. Differential diagnoses of muscle hypertrophy include exercise, denervation, radiation, and myopathies [6]. In our case, the patient has none of these etiologies. 

Moreover, two cases of unilateral TFL hypertrophy were present at Hospital Universitario de Fuenlabrada. Both patients were treated with conservative treatment and clinical observation due to the absence of growth and physical limitation [7]. 

A case reported a 68-year-old man with no relevant medical history presenting with asymptomatic swelling gradually increasing in size. The TFL hypertrophy was secondary to an abductor tendon tear [8].

Many cases reported have described an underlying cause, such as a tear following an injury, radiculopathy, or post-surgery. However, our patient did not have any signs of weakness, and based on her MRI the hypertrophy has been stable since her diagnosis. Given the interval stability over three years, biopsy and surgical intervention were not required. The patient was reassured and kept on regular annual follow-up.

## Conclusions

Tensor fascia lata hypertrophy is a rare entity that may simulate a soft tissue tumor. Patients may present with symptoms such as pain and swelling in the anterolateral proximal thigh area. MRI imaging features, in conjunction with the clinical assessment and regular follow-up would help in reaching the correct diagnosis. To avoid the need for invasive procedures, it is essential to understand the imaging characteristics of tensor fascia lata muscle hypertrophy.

We report a case of tensor fascia lata muscle hypertrophy without an identifiable etiology. Imaging characteristics demonstrated interval stability throughout a three-year period; hence, neither a biopsy nor surgical intervention was required. The patient was reassured by the orthopedic oncology team and is currently undergoing yearly follow-up.
